# Revisiting Platinum Sensitivity in Relapsed Small-Cell Lung Cancer: Outcomes of Platinum-Containing Doublet Chemotherapy in the 3–6 Month Relapse Window

**DOI:** 10.3390/curroncol33080472

**Published:** 2026-08-08

**Authors:** İbrahim Çil, Maral Martin Mıldanoğlu, Yasin Kutlu, Ali Kaan Güren, Murat Sarı, İlker Nihat Ökten, Fatih Atalah, Tuba Baydaş, Cevat İlteriş Kıkılı, Deniz Tural, Ayberk Bayramgil, Gözde Balkaya Aykut, Pembegül Yumuştutan, Eda Erçin, Bekir Doğan, Mesut Yılmaz, Özgür Han, Bünyamin Güney, Sercan Olcar, Hatice Odabaş, Ahmet Bilici, Melike Özçelik

**Affiliations:** 1 Department of Medical Oncology, Ümraniye Training and Research Hospital, Istanbul 34764, Türkiye; ayberkbayramgil@gmail.com (A.B.); gozdebalkayaa@gmail.com (G.B.A.); yumustutan@gmail.com (P.Y.); eda.ercin.ee@gmail.com (E.E.); drmelike.ozcelik@gmail.com (M.Ö.); 2 Department of Medical Oncology, Faculty of Medicine, Medipol University, Istanbul 34214, Türkiye; mmildanoglu@gmail.com (M.M.M.); dryasinkutlu@gmail.com (Y.K.); ahmetknower@yahoo.com (A.B.); 3 Department of Medical Oncology, School of Medicine, Marmara University, Istanbul 34899, Türkiye; alikaanguren@gmail.com (A.K.G.); drmuratsari@gmail.com (M.S.); 4 Department of Medical Oncology, Göztepe Prof. Dr. Süleyman Yalçın City Hospital, Istanbul 34722, Türkiye; ilkernihat@gmail.com (İ.N.Ö.); fatihatalah@gmail.com (F.A.); tuba.baydas@gmail.com (T.B.); 5 Department of Medical Oncology, School of Medicine, Koç University, Istanbul 34010, Türkiye; ilteris_39@hotmail.com (C.İ.K.); deniztural@gmail.com (D.T.); 6 Department of Medical Oncology, Bakırköy Dr. Sadi Konuk Training and Research Hospital, Istanbul 34147, Türkiye; bekirgata46@gmail.com (B.D.); mesutyilmaz12@yahoo.com (M.Y.); 7 Department of Medical Oncology, Istanbul Training and Research Hospital, Istanbul 34098, Türkiye; ozgurhan@gmail.com (Ö.H.); drbunyaminguney01@gmail.com (B.G.); 8 Department of Medical Oncology, Kartal Dr. Lütfi Kırdar City Hospital, Istanbul 34865, Türkiye; sercan.olcar@hotmail.com (S.O.); odabashatice@yahoo.com (H.O.)

**Keywords:** small-cell lung cancer, platinum rechallenge, platinum-free interval, second-line chemotherapy

## Abstract

**Background:** Second-line treatment selection in relapsed extensive-stage small-cell lung cancer (ES-SCLC) is commonly guided by the platinum-free interval, but the optimal approach for patients progressing 3–6 months after first-line platinum-based therapy remains uncertain. We compared platinum-containing doublet chemotherapy with single-agent chemotherapy in this clinically ambiguous subgroup. **Methods:** This multicenter retrospective real-world cohort study included patients with ES-SCLC or recurrent metastatic SCLC after prior limited-stage disease who received first-line platinum-based chemotherapy, achieved disease control, and progressed within a platinum-free interval of 90–180 days. Treatment allocation was at the discretion of the treating physician and was not randomized. The primary endpoint was progression-free survival (PFS); secondary endpoints were overall survival (OS), objective response rate (ORR), disease control rate (DCR), and safety. Because of the non-randomized design, the treatment effect was examined across multiple analytic approaches, including multivariable Cox regression, propensity score adjustment, and stabilized inverse probability of treatment weighting (IPTW). **Results:** Of 105 patients, 54 received single-agent chemotherapy and 51 received platinum-containing doublet therapy; 39 (37.1%) had received first-line atezolizumab. Baseline characteristics were imbalanced in favor of the doublet group, which had a longer platinum-free interval, fewer pleural metastases, and a higher rate of objective response to first-line therapy. ORR was higher with doublet therapy (31.4% vs. 11.1%, *p* = 0.016), as was DCR (62.7% vs. 33.3%, *p* = 0.003). Median PFS was 4.4 months (95% CI 3.4–5.8) with doublet therapy versus 3.1 months (95% CI 2.7–3.7) with single-agent chemotherapy (unadjusted HR 0.53, 95% CI 0.36–0.80; *p* = 0.002). Median OS was 6.5 months (95% CI 5.4–8.0) versus 5.4 months (95% CI 4.1–6.0), a difference that was not statistically significant (HR 0.68, 95% CI 0.46–1.01; *p* = 0.054). The PFS estimate favored doublet therapy in all sensitivity analyses but was attenuated with increasingly complete adjustment for treatment selection (multivariable HR 0.46, 95% CI 0.29–0.72; propensity-adjusted HR 0.58, 95% CI 0.38–0.88; IPTW HR 0.68, 95% CI 0.38–1.20, *p* = 0.184). No OS estimate reached statistical significance in any model. Grade ≥ 3 adverse events were similar between groups (66.7% vs. 64.8%, *p* = 0.842). **Conclusions:** In this non-randomized cohort of patients relapsing within a 90–180-day platinum-free interval, platinum-containing doublet chemotherapy was associated with higher response rates and longer PFS, without an increase in severe toxicity, but no overall survival benefit was demonstrated. Because baseline prognostic factors consistently favored the doublet group and the PFS advantage was attenuated after propensity-based adjustment, these findings should be regarded as hypothesis-generating. They are nonetheless consistent with randomized data showing improved PFS but not OS with platinum rechallenge, and support platinum-containing rechallenge as a reasonable option in carefully selected patients rather than as a demonstrated survival-prolonging strategy.

## Simple Summary

Patients with small-cell lung cancer almost always relapse after their first chemotherapy treatment. When relapse occurs more than six months after treatment, doctors usually feel comfortable using platinum-based chemotherapy again; when it occurs within three months, they usually do not. Patients who relapse between three and six months fall into an uncertain middle ground, and there is little evidence to guide treatment. We reviewed the records of 105 such patients treated at eight centers in Türkiye. Patients who received a platinum-based two-drug combination had better tumor shrinkage and a longer time before their disease worsened than patients who received a single drug, with a similar rate of serious side effects. However, they did not live significantly longer, and the patients selected for the two-drug combination had more favorable characteristics to begin with, which may explain part of the difference. Our findings suggest that platinum-based combination chemotherapy remains a reasonable option for carefully chosen patients in this three-to-six month window, but that it should not be assumed to prolong survival.

## 1. Introduction

Small-cell lung cancer (SCLC) is an aggressive neuroendocrine malignancy that accounts for approximately 12–15% of all lung cancers and is characterized by rapid tumor growth, early metastatic dissemination, high symptom burden, and poor survival outcomes [1,2]. Most patients present with extensive-stage disease, and although SCLC is initially highly sensitive to cytotoxic chemotherapy, relapse after first-line treatment remains almost inevitable [3]. Consequently, the management of relapsed extensive-stage SCLC (ES-SCLC) continues to represent a major clinical challenge.

For decades, platinum-etoposide chemotherapy was the standard first-line treatment for ES-SCLC. More recently, the addition of immune checkpoint inhibition to platinum-etoposide has changed the therapeutic landscape. The phase III IMpower133 trial demonstrated an overall survival benefit with atezolizumab plus carboplatin-etoposide compared with chemotherapy alone, while the phase III CASPIAN trial showed improved survival with durvalumab plus platinum-etoposide compared with platinum-etoposide alone [4,5]. Based on these studies, platinum-etoposide combined with a PD-L1 inhibitor has become the preferred first-line standard for eligible patients with ES-SCLC [6].

Despite the incorporation of immunotherapy into first-line treatment, most patients eventually experience disease progression, and treatment options in the second-line setting remain limited. Historically, second-line treatment selection has been guided largely by the quality of response to first-line therapy and by the interval between completion of platinum-based chemotherapy and relapse. Patients with early relapse are generally considered to have platinum-refractory or resistant disease, whereas those relapsing after a longer interval are considered to have platinum-sensitive disease and may be candidates for platinum rechallenge [6,7].

However, the optimal cutoff defining platinum sensitivity remains debated. Several studies and guidelines have used a 90-day threshold after completion of first-line chemotherapy to define sensitive relapse, and this definition has been adopted in prospective and retrospective studies evaluating platinum rechallenge [8,9]. In the randomized phase III trial by Baize et al., carboplatin-etoposide rechallenge significantly prolonged progression-free survival compared with topotecan in patients with sensitive relapsed SCLC, supporting platinum rechallenge as a reasonable option in selected patients [9]. Similarly, retrospective studies have reported meaningful activity of platinum-based rechallenge in patients with platinum-sensitive relapse [8,10].

Nevertheless, patients who relapse between 3 and 6 months after first-line platinum-based therapy represent a clinically important gray zone. Although these patients meet the commonly used ≥90-day definition of platinum-sensitive disease, many clinicians remain uncertain whether platinum-containing combination chemotherapy provides sufficient additional benefit compared with single-agent chemotherapy, particularly considering toxicity and patient frailty in the relapsed setting. This uncertainty has become even more relevant in the chemoimmunotherapy era, because the predictive value of traditional platinum-free interval cutoffs may differ after first-line PD-L1 inhibitor exposure [10,11].

Therefore, we conducted a multicenter real-world retrospective study to evaluate the efficacy and safety of platinum-containing doublet chemotherapy compared with single-agent chemotherapy in patients with ES-SCLC or recurrent metastatic SCLC who developed disease progression within a 90–180 day platinum-free interval after first-line platinum-based therapy. This study specifically aimed to clarify whether platinum-containing doublet therapy provides meaningful clinical benefit in the 3–6 month relapse window.

## 2. Methods

### 2.1. Study Design and Patient Population

This was a multicenter, retrospective, real-world cohort study evaluating second-line treatment outcomes in patients with extensive-stage small-cell lung cancer (ES-SCLC) or recurrent metastatic small-cell lung cancer after prior limited-stage disease.

Patients were eligible if they had histologically or cytologically confirmed small-cell lung cancer, received first-line platinum-based chemotherapy for extensive-stage or recurrent metastatic disease, achieved disease control with first-line treatment, and subsequently developed disease progression within a platinum-free interval of 90–180 days. The platinum-free interval was defined as the time from the last dose of first-line platinum-containing chemotherapy to the date of documented disease progression.

Patients were included if they received second-line systemic therapy after progression. Patients with missing essential dates required to calculate the platinum-free interval, progression-free survival, or overall survival were excluded from the relevant analyses. Patients who had initially presented with limited-stage disease were eligible if they subsequently developed recurrent metastatic disease and received platinum-based systemic therapy in the extensive-stage setting.

### 2.2. Treatment Groups

The main comparison was performed according to the type of second-line systemic treatment. Patients were categorized into two groups:

1. Single-agent chemotherapy group, including patients treated with non-platinum monotherapy, topotecan or irinotecan.
2. Platinum-containing doublet group, including patients treated with platinum-based combination chemotherapy, such as carboplatin or cisplatin combined with etoposide or irinotecan.

First-line treatment consisted of platinum-etoposide chemotherapy with or without atezolizumab. First-line immunotherapy exposure was recorded separately and used for subgroup and adjusted analyses.

#### 2.2.1. Treatment Allocation in Routine Practice

Treatment allocation was not randomized and was not governed by any protocol or institutional algorithm. Second-line regimen selection was made by the treating physician at each participating center. In routine practice at these centers, the factors most commonly weighed were performance status and expected tolerability, the duration of the platinum-free interval within the 90–180 day window, the depth and durability of response to first-line platinum-based therapy, residual hematological and non-hematological toxicity from first-line treatment, bone marrow and organ reserve, comorbidity burden, symptom burden and pace of clinical deterioration, patient preference, and local drug availability and reimbursement status. Physicians were also influenced by their subjective assessment of how well an individual patient had tolerated first-line platinum, a factor that is documented inconsistently in medical records and could not be captured as a study variable. Consequently, patients judged less likely to tolerate or benefit from re-exposure to platinum were preferentially treated with single-agent chemotherapy. This is a non-random, prognostically relevant allocation process, and it is the principal source of confounding by indication in this study.

#### 2.2.2. Rationale for the Regimens Studied

The regimens analyzed reflect routine practice in Türkiye rather than a pre-specified selection. Within the platinum-containing doublet group, platinum–irinotecan was the most frequently used combination (38 of 51 patients, 74.5%); platinum–irinotecan is an established regimen in SCLC following the JCOG 9511 trial and is widely used in this setting in our practice environment, particularly in patients previously treated with platinum–etoposide, in whom re-exposure to the same doublet is often considered less attractive. Within the single-agent group, irinotecan monotherapy was used in 26 of 54 patients (48.1%) alongside topotecan in 28 (51.9%), reflecting variable local availability and reimbursement of topotecan.

Several agents used elsewhere in relapsed SCLC could not be evaluated. Amrubicin is not licensed in Türkiye and was not available to any participating center. Tarlatamab and lurbinectedin were not reimbursed and were not accessible in routine practice during the study period. The cohort therefore reflects a health care setting in which chemotherapy remains the only realistic second-line option for most patients, which is the setting in which the present question is most pressing, but which also limits generalizability to health care systems where these agents are routinely available.

### 2.3. Data Collection

Clinical data were obtained retrospectively from institutional medical records. The collected variables included demographic characteristics, smoking history, date of diagnosis, stage at diagnosis, ECOG performance status, metastatic sites at diagnosis, use of prophylactic cranial irradiation, prior limited-stage treatment history, first-line extensive-stage systemic treatment, first-line immunotherapy exposure, first-line treatment start and end dates, number of first-line chemotherapy cycles, date of first-line progression, and best response to first-line treatment.

Before second-line treatment, ECOG performance status and the location and number of metastatic sites were recorded. Second-line treatment regimen, treatment type, treatment start date, progression date, treatment end date, best response to second-line therapy, treatment delays, grade 3 or higher adverse events, specific hematologic and non-hematologic toxicities, toxicity-related treatment discontinuation, date of death, and date of last follow-up were also collected.

### 2.4. Outcomes

The primary endpoint was progression-free survival after second-line therapy. Progression-free survival was defined as the time from second-line treatment initiation to documented disease progression or death from any cause, whichever occurred first. Patients without progression or death were censored at the date of last follow-up.

Secondary endpoints included overall survival, objective response rate, disease control rate, and treatment safety. Overall survival was defined as the time from second-line treatment initiation to death from any cause. Patients who were alive at the time of last follow-up were censored on the last known follow-up date.

Objective response rate was defined as the proportion of patients achieving complete response or partial response as best response to second-line therapy. Disease control rate was defined as the proportion of patients achieving complete response, partial response, or stable disease. Response assessments were based on routine radiologic evaluations performed in clinical practice and recorded by the treating centers.

Safety outcomes included grade 3 or higher adverse events, grade 3 or higher anemia, thrombocytopenia, neutropenia, febrile neutropenia, emesis, stomatitis, diarrhea, treatment delays, and toxicity-related treatment discontinuation.

### 2.5. Statistical Analysis

Descriptive statistics were used to summarize baseline demographic and clinical characteristics. Categorical variables were presented as frequencies and percentages. Continuous variables were presented as median and interquartile range or mean and standard deviation, depending on distribution.

Baseline characteristics were compared between the single-agent chemotherapy group and the platinum-containing doublet group using the chi-square test or Fisher’s exact test for categorical variables, and Student’s *t*-test or Mann–Whitney U test for continuous variables, as appropriate.

Progression-free survival and overall survival were estimated using the Kaplan–Meier method and compared between treatment groups using the log-rank test. Median survival times with 95% confidence intervals were reported. Cox proportional hazards regression models were used to estimate hazard ratios and 95% confidence intervals for progression-free survival and overall survival.

Multivariable Cox proportional hazards models were constructed for progression-free survival and overall survival. All covariates were pre-specified on clinical grounds and entered into the model simultaneously; no stepwise, automated, or significance-based variable selection was performed. The covariates were: second-line treatment group, age (per 10-year increase), sex, ECOG performance status before second-line treatment, number of metastatic sites before second-line treatment, first-line atezolizumab exposure, best response to first-line treatment (complete or partial response versus stable disease), platinum-free interval (per 30-day increase), and the presence of liver and brain metastasis before second-line treatment. With 103 progression events and 101 deaths, the models included approximately 10 events per variable.

The proportional hazards assumption was tested for every covariate using scaled Schoenfeld residuals.

Because treatment allocation was non-random, the robustness of the treatment effect was examined across a pre-specified series of models. A propensity score for receipt of platinum-containing doublet therapy was estimated by logistic regression including age, sex, ECOG performance status, number of metastatic sites, first-line atezolizumab exposure, best response to first-line treatment, platinum-free interval, and the presence of liver, brain, pleural, bone, lymph node and lung metastasis before second-line treatment. Covariate balance before and after weighting was assessed using standardized mean differences, with values below 0.10 considered to indicate adequate balance. The propensity score was used both as a covariate in a Cox model and to derive stabilized inverse probability of treatment weights, which were truncated at the 1st and 99th percentiles; weighted models used robust variance estimation. Additional sensitivity analyses were performed adding pleural metastasis to the primary model, using a parsimonious model restricted to covariates associated with outcome, restricting the cohort to patients with an objective response to first-line therapy, and restricting the cohort to patients with ECOG performance status 0–1. E-values were calculated to quantify the strength of unmeasured confounding that would be required to explain the observed associations.

Subgroup analyses of the treatment effect were performed according to first-line atezolizumab exposure, best response to first-line treatment, platinum-free interval (90–132 versus 133–180 days), ECOG performance status, presence of liver or brain metastasis, and age, with heterogeneity assessed by treatment-by-subgroup interaction terms. Patients who had not progressed or died were censored at the date of last follow-up; the data cut-off was 11 June 2026.

All statistical tests were two-sided, and a *p* value below 0.05 was considered statistically significant. Statistical analyses were performed using IBM SPSS Statistics for Windows, Version 26.0 (IBM Corp., Armonk, NY, USA).

### 2.6. Ethical Considerations

The study was conducted in accordance with the principles of the Declaration of Helsinki. The study protocol was approved by the Umraniye Training and Research Hospital Scientific Research Ethics Committee (Decision No.: 138, approval date: 25 March 2026). Because of the retrospective, non-interventional nature of the study, the requirement for informed consent was waived by the ethics committee.

## 3. Results

### 3.1. Patient Selection and Study Cohort

A total of 748 all-comer patients with small-cell lung cancer were screened across participating centers. Among them, 583 patients (78.0%) received first-line systemic treatment, whereas 165 patients (22.0%) did not receive first-line therapy. Of the patients who received first-line treatment, 350 patients (60.0%) subsequently received second-line systemic therapy, while 233 patients (40.0%) did not proceed to second-line treatment.

**Figure 1:**
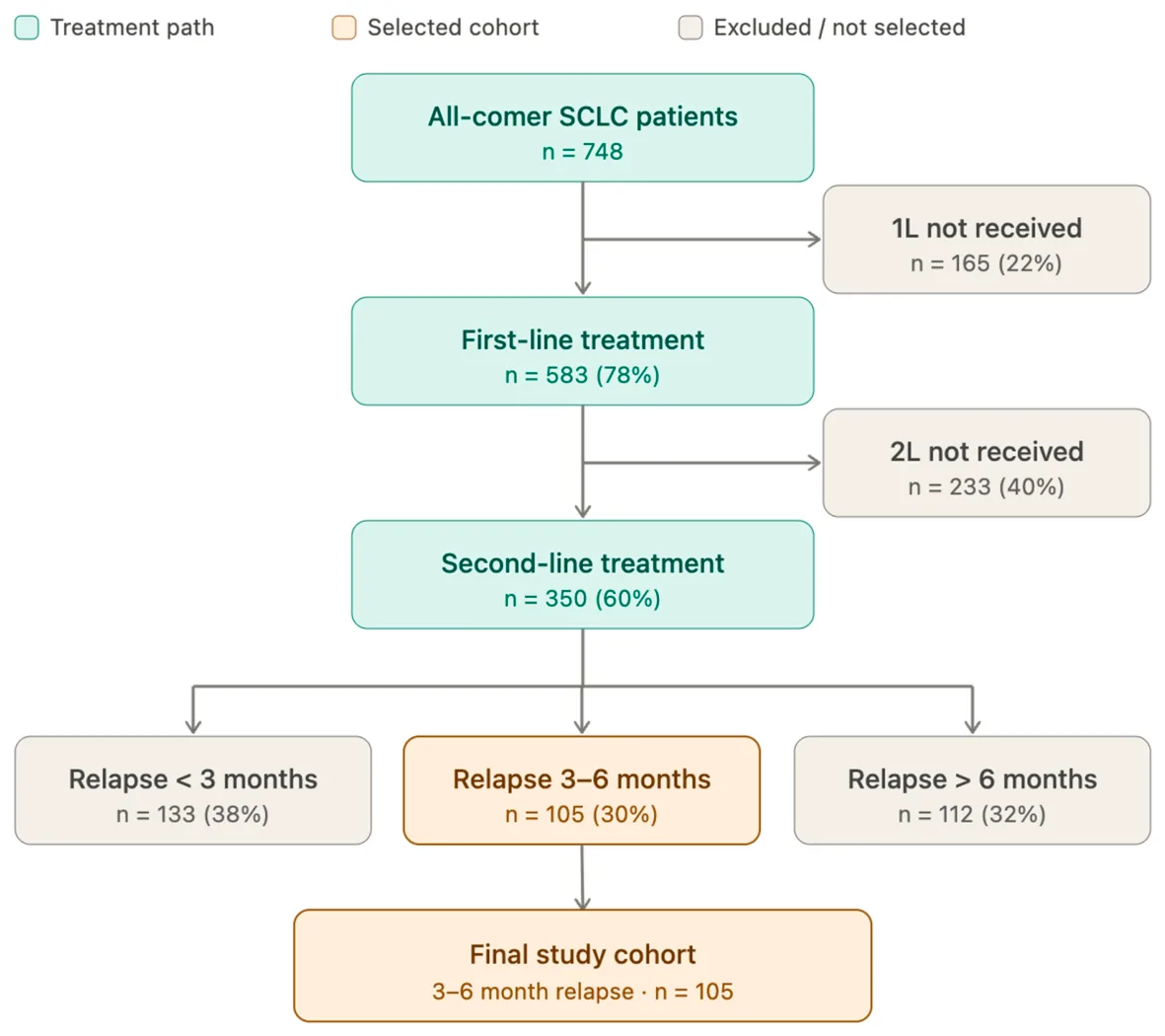
Study flow diagram. The final study cohort included patients with relapse within the 3–6-month platinum-free interval after first-line platinum-based treatment. Abbreviations: 1L, first-line; 2L, second-line; SCLC, small-cell lung cancer.

Among the 350 patients who received second-line therapy, relapse occurred within less than 3 months in 133 patients (38.0%), between 3 and 6 months in 105 patients (30.0%), and after more than 6 months in 112 patients (32.0%). The final study cohort consisted of the 105 patients who developed disease progression within the 3–6 month platinum-free interval and received second-line systemic therapy (Figure 1).

### 3.2. Baseline Characteristics

Baseline demographic and clinical characteristics are summarized in Table 1. The median age at second-line treatment initiation was 64.1 years. Most patients were male (74.3%) and had de novo extensive-stage disease at initial diagnosis (80.0%), whereas 21 patients (20.0%) had initially limited-stage disease and later developed recurrent metastatic disease.

Before second-line treatment initiation, most patients had preserved performance status, with ECOG performance status 0–1 in 91 patients (86.7%). First-line treatment consisted of carboplatin-etoposide in 33 patients (31.4%), cisplatin-etoposide in 33 patients (31.4%), and carboplatin-etoposide plus atezolizumab in 39 patients (37.1%). The best response to first-line treatment was complete response in 10 patients (9.5%), partial response in 78 patients (74.3%), and stable disease in 17 patients (16.2%).

The median platinum-free interval was 132 days. At the time of second-line treatment initiation, the median number of metastatic sites was 3.0. The most frequent metastatic sites before second-line treatment were lymph nodes (74.3%), lung (63.8%), bone (61.9%), brain (41.0%), and liver (38.1%).

Overall, baseline characteristics were generally balanced between the single-agent chemotherapy and platinum-containing doublet groups. Pleural metastasis before second-line treatment was more frequent in the single-agent chemotherapy group than in the platinum-containing doublet group.

### 3.3. Second-Line Treatment and Efficacy Outcomes

Second-line treatment regimens and efficacy outcomes are summarized in Table 2. In the single-agent chemotherapy group, 28 patients (51.9%) received topotecan and 26 patients (48.1%) received irinotecan. In the platinum-containing doublet group, the most frequently used regimen was carboplatin-irinotecan in 33 patients (64.7%), followed by carboplatin-etoposide in 10 patients (19.6%), cisplatin-irinotecan in 5 patients (9.8%), and cisplatin-etoposide in 3 patients (5.9%).

No complete response was observed after second-line treatment. Partial response was achieved in 6 patients (11.1%) in the single-agent chemotherapy group and in 16 patients (31.4%) in the platinum-containing doublet group. The objective response rate was significantly higher with platinum-containing doublet therapy than with single-agent chemotherapy (31.4% vs. 11.1%, *p* = 0.016). Disease control was also more frequent in the platinum-containing doublet group than in the single-agent chemotherapy group (62.7% vs. 33.3%, *p* = 0.003).

**Figure 2:**
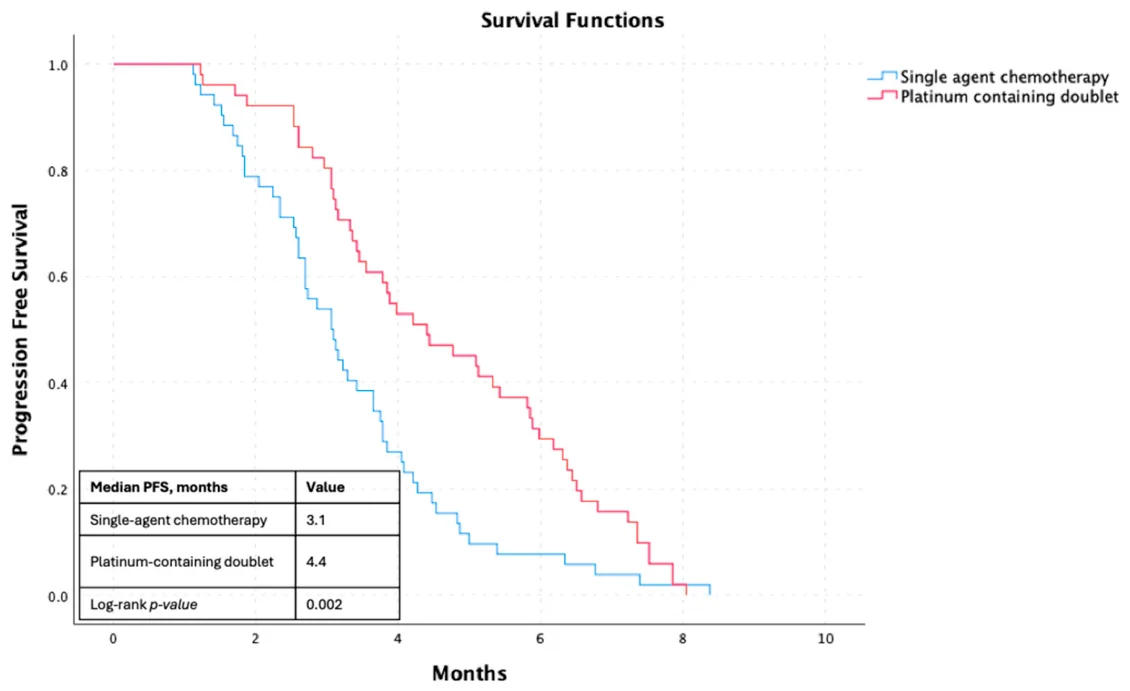
Kaplan–Meier analysis of progression-free survival according to second-line treatment group.

With follow-up calculated from second-line treatment initiation, median progression-free survival was 3.1 months in the single-agent chemotherapy group and 4.4 months in the platinum-containing doublet group. In the Kaplan–Meier analysis, progression-free survival was significantly longer with platinum-containing doublet therapy than with single-agent chemotherapy (HR 0.53, 95% CI 0.36–0.79; log-rank *p* = 0.002) (Figure 2).

**Figure 3:**
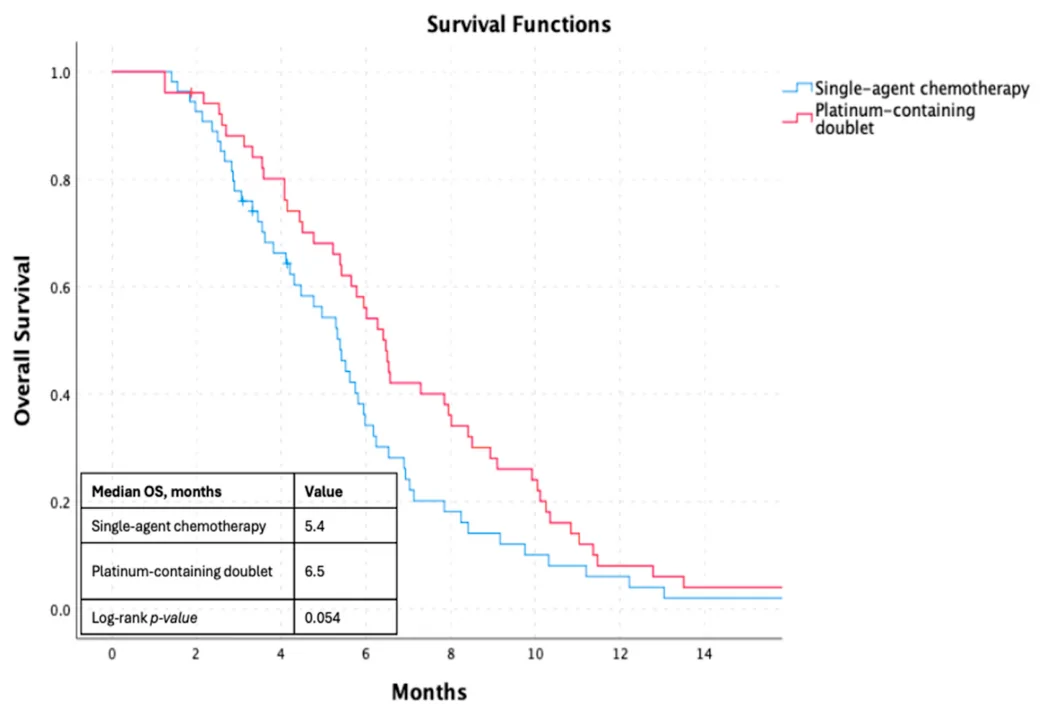
Kaplan–Meier analysis of overall survival according to second-line treatment group.

Median overall survival was 5.4 months in the single-agent chemotherapy group and 6.5 months in the platinum-containing doublet group; this difference showed a favorable trend but did not reach statistical significance (log-rank *p* = 0.054) (Figure 3).

At the data cut-off of 11 June 2026, follow-up was essentially complete: 103 of 105 patients (98.1%) had experienced progression or death and 101 patients (96.2%) had died. Median progression-free survival was 3.1 months (95% CI 2.7–3.7) in the single-agent group and 4.4 months (95% CI 3.4–5.8) in the platinum-containing doublet group. Median overall survival was 5.4 months (95% CI 4.1–6.0) and 6.5 months (95% CI 5.4–8.0), respectively.

### 3.4. Safety Outcomes

Safety outcomes during second-line treatment are summarized in Table 3. Overall, grade 3 or higher adverse events were common in both groups and were reported in 69 patients (65.7%). The frequency of any grade 3 or higher adverse event was similar between the single-agent chemotherapy and platinum-containing doublet groups (64.8% vs. 66.7%, *p* = 0.842).

The most frequent grade 3 or higher toxicity in the overall cohort was neutropenia, observed in 46 patients (43.8%), followed by thrombocytopenia in 32 patients (30.5%) and anemia in 26 patients (24.8%). Grade 3 or higher neutropenia occurred in 48.1% of patients receiving single-agent chemotherapy and 39.2% of patients receiving platinum-containing doublet therapy. Grade 3 or higher thrombocytopenia was numerically more frequent in the single-agent chemotherapy group than in the platinum-containing doublet group, although the difference did not reach statistical significance (38.9% vs. 21.6%, *p* = 0.054).

Non-hematologic grade 3 or higher toxicities were less frequent. Grade 3 or higher diarrhea occurred in 13.3% of the overall cohort, febrile neutropenia in 8.6%, emesis in 5.7%, and stomatitis in 1.9%. Toxicity-related treatment discontinuation was infrequent and occurred in 4 patients (7.4%) in the single-agent chemotherapy group and 3 patients (5.9%) in the platinum-containing doublet group.

### 3.5. Cox Regression Analyses for Survival Outcomes

Univariable and multivariable Cox regression analyses for progression-free survival and overall survival are shown in Table 4 and Table 5. In univariable analysis, platinum-containing doublet therapy was associated with significantly longer progression-free survival compared with single-agent chemotherapy (HR 0.53, 95% CI 0.36–0.79; *p* = 0.002). Higher ECOG performance status, liver metastasis, and brain metastasis before second-line treatment were associated with shorter progression-free survival.

After adjustment for clinically relevant baseline factors, platinum-containing doublet therapy remained independently associated with longer progression-free survival (adjusted HR 0.45, 95% CI 0.29–0.71; *p* < 0.001). Higher ECOG performance status before second-line treatment was also independently associated with shorter progression-free survival.

For overall survival, platinum-containing doublet therapy was associated with a numerically lower risk of death in univariable analysis, although this association did not reach conventional statistical significance (HR 0.68, 95% CI 0.46–1.01; *p* = 0.054). Higher ECOG performance status, liver metastasis, and brain metastasis before second-line treatment were associated with shorter overall survival in univariable analysis. In the multivariable model, ECOG performance status and liver metastasis remained independently associated with worse overall survival, whereas platinum-containing doublet therapy showed a favorable but non-significant trend (adjusted HR 0.67, 95% CI 0.44–1.03; *p* = 0.068).

### 3.6. Sensitivity and Propensity Score Analyses

Because treatment allocation was non-random, the treatment effect was examined across eight analytic approaches (Table 6). Baseline covariates were imbalanced between groups before weighting, and the imbalance consistently favored the platinum-containing doublet group: standardized mean differences exceeded 0.10 for pleural metastasis (−0.50), platinum-free interval (+0.41), objective response to first-line treatment (+0.34), lymph node metastasis (−0.34), number of metastatic sites (−0.34), bone metastasis (−0.28), ECOG performance status (−0.23), brain metastasis (−0.15) and age (+0.14). The propensity score model had a c-statistic of 0.753, and after stabilized inverse probability of treatment weighting, all standardized mean differences fell below 0.10, indicating adequate balance.

The direction of the treatment effect on progression-free survival favored platinum-containing doublet therapy in every model. However, the magnitude of the effect was progressively attenuated as adjustment for treatment selection became more complete, from an unadjusted hazard ratio of 0.53 (95% CI 0.36–0.80) to 0.58 (95% CI 0.38–0.88) with propensity score adjustment and 0.68 (95% CI 0.38–1.20; *p* = 0.184) under inverse probability of treatment weighting, where the association was no longer statistically significant. Similar estimates were obtained with alternative propensity model specifications and without weight truncation (progression-free survival hazard ratios 0.63–0.68). For overall survival, the hazard ratio was stable across models (range 0.68–0.79) but did not reach statistical significance in any analysis.

For the adjusted progression-free survival estimate, the E-value was 3.77 for the point estimate and 2.12 for the confidence limit, indicating that an unmeasured confounder would need to be associated with both treatment allocation and progression by a hazard ratio of at least 2.12, beyond all measured covariates, to render the association non-significant. For overall survival, the E-value for the confidence limit was 1.00, indicating that no unmeasured confounding is required to account for the absence of a significant survival difference.

The proportional hazards assumption was satisfied for all covariates in the progression-free survival model. In the overall survival model, the assumption was violated for age (*p* = 0.01); an overall survival model stratified by age category (<65 versus ≥65 years) yielded an essentially unchanged treatment estimate (HR 0.72, 95% CI 0.46–1.13).

### 3.7. Subgroup Analyses

**Figure 4:**
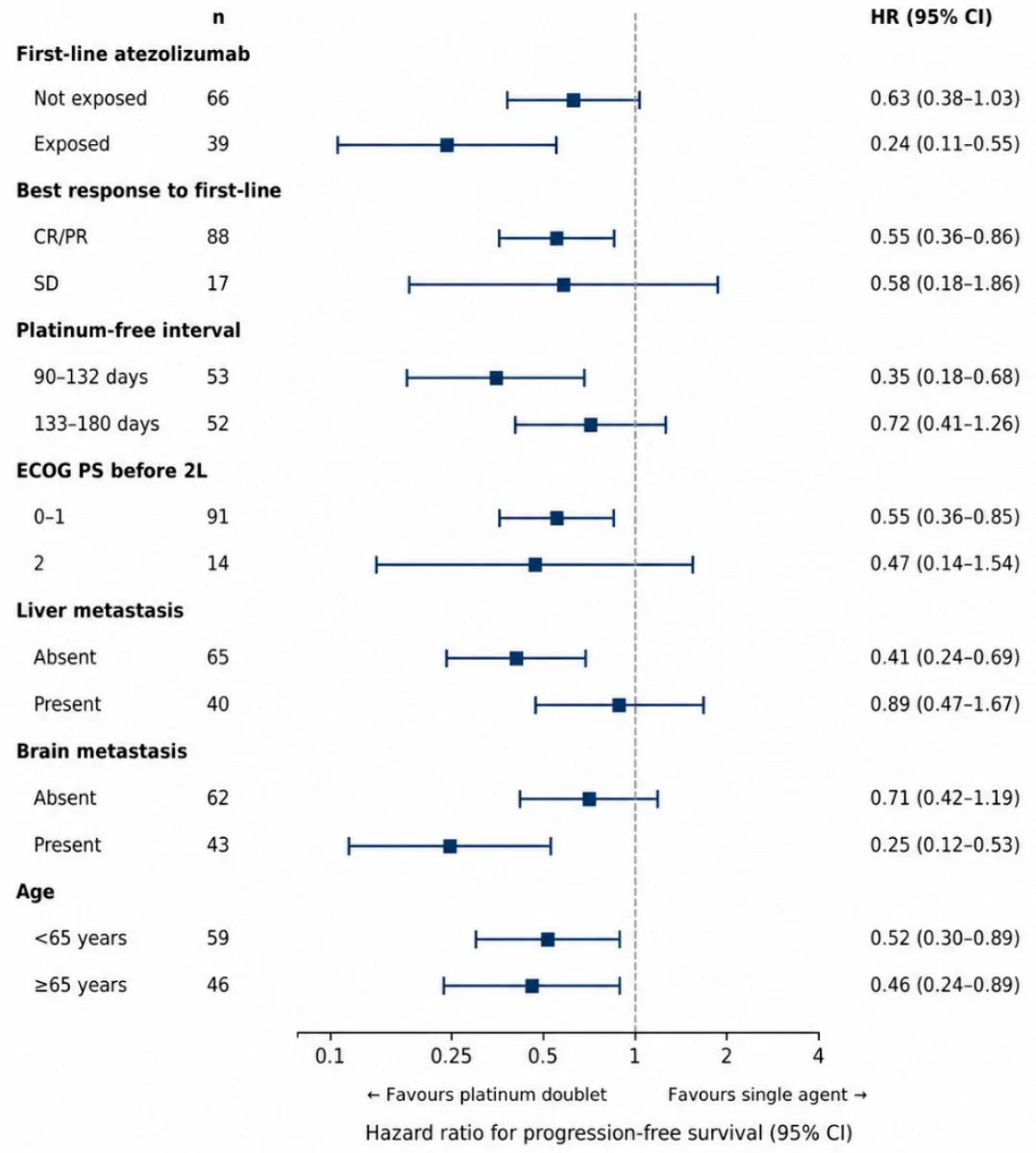
Forest plot of progression-free survival subgroup analyses according to second-line treatment group.

Subgroup analyses of progression-free survival are shown in Figure 4. The direction of effect favored platinum-containing doublet therapy in all examined subgroups, and no test for interaction was statistically significant for either progression-free survival or overall survival (all *p* > 0.10). Among the 39 patients exposed to first-line atezolizumab, the progression-free survival hazard ratio was 0.24 (95% CI 0.11–0.55), compared with 0.63 (95% CI 0.38–1.03) in the 66 patients without first-line immunotherapy exposure; however, the interaction test was not significant (*p* = 0.106) and both subgroups were small. Given the number of comparisons and the limited size of individual subgroups, these analyses are exploratory and should not be used to guide treatment selection.

## 4. Discussion

In this multicenter real-world cohort of patients with extensive-stage or recurrent metastatic SCLC who progressed within a 90–180-day platinum-free interval after first-line platinum-based therapy, second-line platinum-containing doublet chemotherapy was associated with a higher objective response rate, a higher disease control rate, and longer progression-free survival than single-agent chemotherapy, without an increase in grade 3 or higher toxicity or toxicity-related treatment discontinuation. Overall survival was numerically longer with doublet therapy, but the difference did not reach statistical significance in any of the models we examined. Importantly, treatment allocation was non-random and baseline prognostic factors consistently favored the doublet group, and the progression-free survival advantage was attenuated and lost statistical significance after inverse probability of treatment weighting. These findings should therefore be interpreted as hypothesis-generating. They suggest that patients relapsing within the 3–6 month interval should not be regarded as uniformly and functionally platinum-resistant, and that platinum-containing doublet therapy remains a reasonable option in carefully selected patients, but they do not establish that this strategy prolongs survival.

The clinical relevance of this question derives from the long-standing uncertainty surrounding the definition of platinum sensitivity in relapsed SCLC. Traditionally, patients with early relapse have been considered platinum-refractory or platinum-resistant, whereas those relapsing after a longer treatment-free interval have been considered platinum-sensitive [1,2,3]. However, the cutoff used to define platinum sensitivity has varied across studies and clinical practice. A 90-day threshold after completion of first-line chemotherapy is commonly used in trials and guidelines, but many clinicians have historically regarded relapse after more than 6 months as a more convincing marker of platinum sensitivity [6,12,13]. Therefore, the 3–6 month interval represents a clinically important gray zone in which the optimal choice between platinum rechallenge and non-platinum single-agent therapy remains uncertain. This ambiguity is further supported by recent work showing that traditional 60- or 90-day cutoffs were not derived from rigorous analytical methods and may not fully reflect outcomes in the contemporary immunotherapy era [11].

Our findings are consistent with earlier retrospective studies suggesting that platinum rechallenge may benefit selected patients with platinum-sensitive relapsed SCLC. In a Turkish multicenter retrospective analysis, Korkmaz et al. reported that, among platinum-sensitive patients, platinum-based rechallenge was associated with higher response rate, longer progression-free survival, and longer overall survival compared with non-platinum therapy [3]. Similarly, Genestreti et al. evaluated platinum/etoposide rechallenge in patients with platinum-sensitive SCLC and reported a median progression-free survival of 5.5 months and median overall survival from rechallenge of 7.9 months, concluding that rechallenge was feasible and clinically reasonable in this setting [14]. Other retrospective series have also supported the activity of rechallenge-based strategies in sensitive relapse, although patient selection, relapse interval definitions, and treatment regimens differed across studies [8,15].

The strongest prospective evidence supporting platinum rechallenge comes from the randomized phase III trial by Baize et al., in which carboplatin-etoposide significantly improved progression-free survival compared with topotecan in patients with sensitive relapsed SCLC [9]. In that study, eligible patients had responded to first-line platinum-etoposide and relapsed at least 90 days after completion of first-line treatment, and median progression-free survival was 4.7 months with carboplatin-etoposide versus 2.7 months with topotecan. Our results are directionally consistent with this trial, although our population was more narrowly focused on the 90–180 day interval and therefore represents a clinically more uncertain subgroup.

It is important to note that the pattern we observed closely mirrors that of the only randomized trial in this setting. In the trial by Baize et al., carboplatin–etoposide rechallenge improved objective response rate (49% versus 25%) and progression-free survival (4.7 versus 2.7 months, HR 0.57) compared with topotecan, yet median overall survival was virtually identical between arms (7.5 versus 7.4 months; HR 1.03, 95% CI 0.87–1.19; *p* = 0.94) [9]. Our real-world data reproduce this dissociation between progression-free and overall survival: a clear advantage in response and progression-free survival, and no demonstrable survival benefit. Several explanations are plausible, including the availability of subsequent lines of therapy, the aggressive natural history of relapsed SCLC after second-line progression, and the limited statistical power of both studies for overall survival. Whatever the mechanism, the consistency between a randomized trial and an independent real-world cohort restricted to the narrower 3–6 month window strengthens the conclusion that platinum rechallenge in this population should be presented to patients as a strategy that may improve disease control and delay progression, rather than as one that has been shown to prolong life.

This distinction is important because several prior studies included all patients with relapse beyond 90 days, thereby combining patients with relatively early relapse and those with much longer relapse-free intervals [9,14]. In contrast, our cohort was restricted to the 3–6 month population, which is precisely the subgroup in which clinical equipoise is greatest. The observed improvement in response and progression-free survival suggests that at least a subset of these patients may retain clinically relevant platinum sensitivity, even though their relapse occurs earlier than the traditional 6-month threshold often used in daily practice.

The relevance of platinum rechallenge has become even more complex in the chemoimmunotherapy era. First-line platinum-etoposide combined with PD-L1 inhibition has become a standard treatment for eligible patients with ES-SCLC after the survival benefits demonstrated in IMpower133 and CASPIAN [4,5]. Nevertheless, most patients still experience disease progression, and the optimal sequencing of second-line therapy after chemoimmunotherapy remains incompletely defined. In a large French multicenter real-world study evaluating second-line treatment after first-line chemotherapy plus immunotherapy, platinum rechallenge was associated with longer survival outcomes compared with lurbinectedin or other chemotherapy regimens [10]. In that study, median overall survival was 8.1 months with platinum rechallenge, compared with 4.9 months with lurbinectedin and 5.1 months with other regimens, while median second-line progression-free survival was 4.6, 2.7, and 2.2 months, respectively. Our results are consistent with these findings and extend them by focusing specifically on the 3–6 month platinum-free interval, a group that remains underrepresented in prospective studies.

Other second-line options should also be considered when interpreting these findings. Topotecan has historically been one of the most established second-line treatments, but its clinical benefit is modest and its hematologic toxicity can be substantial [16]. Lurbinectedin has demonstrated activity in relapsed SCLC and has become an important option in many treatment algorithms, particularly for patients who are not candidates for platinum rechallenge [17]. However, real-world post-immunotherapy data suggest that lurbinectedin outcomes may be less favorable than platinum rechallenge in selected platinum-sensitive patients [10]. Therefore, even in an expanding treatment landscape, the question of whether selected patients can still benefit from platinum-containing chemotherapy remains clinically relevant.

The therapeutic landscape of relapsed ES-SCLC is also evolving with DLL3-targeted therapy. Tarlatamab, a DLL3-directed bispecific T-cell engager, has demonstrated clinically meaningful activity in previously treated ES-SCLC and has emerged as an important option after progression on platinum-based chemotherapy [18,19]. However, access to tarlatamab may be limited in many countries because of cost, reimbursement restrictions, drug availability, and the need for clinical infrastructure capable of monitoring and managing immune-mediated toxicities such as cytokine release syndrome and neurologic adverse events [20]. Therefore, chemotherapy-based strategies remain highly relevant in real-world practice, particularly in health care systems where novel agents are unavailable, delayed, or restricted. In this context, defining which patients within the 3–6 month relapse window may still benefit from platinum-containing doublet therapy remains a practical and clinically meaningful question.

The safety findings of our study are also clinically relevant. One of the main reasons clinicians avoid platinum rechallenge in the 3–6 month setting is concern regarding cumulative hematologic toxicity, treatment delays, and reduced tolerability in a population with rapidly progressive disease and declining performance status [9,16]. In our cohort, grade 3 or higher adverse events were common in both groups, as expected in relapsed SCLC, but their frequency was similar between single-agent chemotherapy and platinum-containing doublet therapy. Toxicity-related treatment discontinuation was also infrequent and not increased with platinum-containing doublet therapy. These findings suggest that, in appropriately selected patients, the efficacy advantage of platinum-containing doublet therapy may not necessarily come at the cost of substantially greater severe toxicity. However, because toxicity data were collected retrospectively from routine clinical records, underreporting remains possible.

From a practical perspective, our findings do not imply that all patients relapsing within 3–6 months should receive platinum rechallenge. Rather, they support a more individualized approach. Platinum-containing doublet therapy may be most appropriate for patients with preserved performance status, adequate marrow and organ function, prior disease control with first-line platinum-based therapy, and no prohibitive residual toxicity from first-line treatment [6,9,13]. Conversely, patients with poor performance status, rapid symptomatic deterioration, severe marrow compromise, major comorbidities, or poor tolerance of first-line platinum may still be better served by single-agent chemotherapy, best supportive care, clinical trial enrollment, or novel agents when accessible [13,16,21]. Therefore, the 3–6 month interval should be viewed not as an automatic indication for or against platinum rechallenge, but as a setting where patient selection is critical.

This study has several limitations. First and most importantly, treatment allocation was non-random and determined by physician judgment, and baseline characteristics differed systematically between groups in a direction that favored the platinum-containing doublet group: these patients had a longer platinum-free interval, a higher rate of objective response to first-line therapy, fewer pleural, lymph node and bone metastases, and better performance status. Several of the factors that drive regimen selection in routine practice—in particular the treating physician’s subjective assessment of how well an individual patient tolerated and responded to first-line platinum, and the physician’s expectation of tolerability on re-exposure—are documented inconsistently in medical records and could not be measured. Residual confounding by indication therefore cannot be excluded, and the attenuation of the progression-free survival effect after propensity-based weighting indicates that at least part of the apparent advantage is attributable to treatment selection. Second, the sample size was modest; with approximately 10 events per variable, the multivariable models were adequately but not generously powered, and subgroup analyses were clearly underpowered. Third, the cohort reflects a transition period in which only 37.1% of patients received first-line atezolizumab, limiting conclusions about the contemporary post-immunotherapy population. Fourth, treatment was heterogeneous within both groups, and the specific regimens used—predominantly platinum–irinotecan among doublets and irinotecan or topotecan among single agents—reflect Turkish practice; amrubicin, tarlatamab and lurbinectedin were unavailable, which limits generalizability to health care systems in which these agents are accessible. Fifth, response assessments were performed in routine clinical practice rather than by blinded centralized radiologic review, and toxicity was abstracted retrospectively from medical records, so adverse events were likely underestimated. Finally, no formal sample size calculation was performed, and the study was not powered to detect a difference in overall survival.

Despite these limitations, the study addresses a clinically important and underexplored question in relapsed SCLC. By focusing specifically on patients with a 90–180-day platinum-free interval, our analysis provides real-world evidence for a population frequently encountered in daily oncology practice but often diluted within broader “platinum-sensitive” cohorts. The findings support the hypothesis that platinum-containing doublet therapy can provide meaningful response and progression-free survival benefit in selected patients within this gray-zone relapse interval, without a clear increase in severe toxicity.

In conclusion, among patients with ES-SCLC or recurrent metastatic SCLC who relapsed 3–6 months after completion of first-line platinum-based therapy, platinum-containing doublet chemotherapy was associated with improved response and progression-free survival compared with single-agent chemotherapy. Overall survival was numerically longer, although the benefit was modest. These results support consideration of platinum-containing rechallenge in carefully selected patients within the 90–180 day platinum-free interval and highlight the need for prospective validation, particularly in the modern chemoimmunotherapy era and in settings where access to novel agents remains limited.

## 5. Conclusions

Among patients with ES-SCLC or recurrent metastatic SCLC relapsing 3–6 months after completion of first-line platinum-based therapy, second-line platinum-containing doublet chemotherapy was associated with higher response rates and longer progression-free survival than single-agent chemotherapy, with a comparable severe toxicity profile. No overall survival benefit was demonstrated, and the progression-free survival advantage was attenuated after adjustment for a non-random and prognostically favorable pattern of treatment allocation. These real-world findings are concordant with randomized evidence showing improved progression-free survival but not overall survival with platinum rechallenge in sensitive relapse. They support platinum-containing doublet chemotherapy as a reasonable and tolerable option for carefully selected patients within the 3–6 month relapse window—particularly those with preserved performance status, good tolerance of first-line platinum, and limited access to novel agents—but they do not justify a routine recommendation for platinum rechallenge in this population. Prospective evaluation is required.

## Figures and Tables

**Table 1:** Baseline demographic and clinical characteristics according to second-line treatment group.

Characteristic	Overall (*n* = 105)	Single-Agent Chemotherapy (*n* = 54)	Platinum-Containing Doublet (*n* = 51)	*p* Value
Age at second-line treatment, years	64.1 (58.0–69.8)	63.2 (58.0–69.7)	65.1 (58.1–70.2)	0.432
Male sex	78 (74.3)	41 (75.9)	37 (72.5)	0.692
De novo extensive-stage disease	84 (80.0)	44 (81.5)	40 (78.4)	0.696
ECOG PS before second-line treatment				0.496
0	12 (11.4)	5 (9.3)	7 (13.7)	
1	79 (75.2)	40 (74.1)	39 (76.5)	
2	14 (13.3)	9 (16.7)	5 (9.8)	
First-line regimen				0.886
Carboplatin-etoposide	33 (31.4)	18 (33.3)	15 (29.4)	
Cisplatin-etoposide	33 (31.4)	17 (31.5)	16 (31.4)	
Carboplatin-etoposide-atezolizumab	39 (37.1)	19 (35.2)	20 (39.2)	
First-line atezolizumab exposure	39 (37.1)	19 (35.2)	20 (39.2)	0.669
Best response to first-line treatment				0.111
CR	10 (9.5)	3 (5.6)	7 (13.7)	
PR	78 (74.3)	39 (72.2)	39 (76.5)	
SD	17 (16.2)	12 (22.2)	5 (9.8)	
Objective response to first-line treatment, CR/PR	88 (83.8)	42 (77.8)	46 (90.2)	0.084
Platinum-free interval, days	132.0 (105.0–157.0)	124.5 (103.2–145.5)	141.0 (112.0–169.5)	0.050
No. metastatic sites before second-line treatment	3.0 (2.0–4.0)	3.0 (2.2–4.0)	3.0 (2.0–4.0)	0.126
Liver metastasis before second-line treatment	40 (38.1)	21 (38.9)	19 (37.3)	0.863
Lung metastasis before second-line treatment	67 (63.8)	34 (63.0)	33 (64.7)	0.853
Bone metastasis before second-line treatment	65 (61.9)	37 (68.5)	28 (54.9)	0.151
Brain metastasis before second-line treatment	43 (41.0)	24 (44.4)	19 (37.3)	0.454
Adrenal metastasis before second-line treatment	21 (20.0)	10 (18.5)	11 (21.6)	0.696
Lymph node metastasis before second-line treatment	78 (74.3)	44 (81.5)	34 (66.7)	0.083
Pleural metastasis before second-line treatment	18 (17.1)	14 (25.9)	4 (7.8)	0.014

Note: Data are presented as median (interquartile range) or *n* (%). Percentages were calculated within the relevant column. The platinum-free interval was defined as the time from completion of first-line platinum-containing chemotherapy to documented disease progression. Metastatic sites refer to disease distribution before initiation of second-line therapy, not sites of progression. *p* values were calculated using the chi-square test or Fisher’s exact test for categorical variables and the Mann–Whitney U test for continuous variables, as appropriate. Abbreviations: CR, complete response; ECOG PS, Eastern Cooperative Oncology Group performance status; PR, partial response; SD, stable disease.

**Table 2:** Second-line treatment regimens and efficacy outcomes according to treatment group.

Variable	Overall (*n* = 105)	Single-Agent Chemotherapy (*n* = 54)	Platinum-Containing Doublet (*n* = 51)	*p* Value
Second-line regimen				—
Topotecan	28 (26.7)	28 (51.9)	0	
Irinotecan	26 (24.8)	26 (48.1)	0	
Carboplatin-irinotecan	33 (31.4)	0	33 (64.7)	
Cisplatin-irinotecan	5 (4.8)	0	5 (9.8)	
Carboplatin-etoposide	10 (9.5)	0	10 (19.6)	
Cisplatin-etoposide	3 (2.9)	0	3 (5.9)	
Best response to second-line treatment				0.006
CR	0	0	0	
PR	22 (21.0)	6 (11.1)	16 (31.4)	
SD	28 (26.7)	12 (22.2)	16 (31.4)	
PD	55 (52.4)	36 (66.7)	19 (37.3)	
Objective response rate, CR/PR	22 (21.0)	6 (11.1)	16 (31.4)	0.016
Disease control rate, CR/PR/SD	50 (47.6)	18 (33.3)	32 (62.7)	0.003
Median PFS, months (95% CI)	3.6 (3.1–4.0)	3.1 (2.7–3.7)	4.4 (3.4–5.8)	0.002
Median OS, months (95% CI)	5.8 (5.2–6.4)	5.4 (4.1–6.0)	6.5 (5.4–8.0)	0.054

Note: Data are presented as *n* (%) unless otherwise specified. Percentages were calculated within the relevant column. Objective response rate was defined as complete response or partial response. Disease control rate was defined as complete response, partial response, or stable disease. PFS was defined as the time from second-line treatment initiation to progression or death from any cause, whichever occurred first. OS was defined as the time from second-line treatment initiation to death from any cause. The *p* value for best response distribution was calculated using the chi-square test. The *p* values for ORR and DCR were calculated using Fisher’s exact test. *p* values for PFS and OS were obtained from univariable Cox regression analysis comparing platinum-containing doublet therapy with single-agent chemotherapy. Abbreviations: CR, complete response; DCR, disease control rate; ORR, objective response rate; OS, overall survival; PD, progressive disease; PFS, progression-free survival; PR, partial response; SD, stable disease.

**Table 3:** Safety outcomes according to second-line treatment group.

Safety Outcome	Overall (*n* = 105)	Single-Agent Chemotherapy (*n* = 54)	Platinum-Containing Doublet (*n* = 51)	*p* Value
Any grade ≥ 3 adverse event	69 (65.7)	35 (64.8)	34 (66.7)	0.842
Grade ≥ 3 anemia	26 (24.8)	11 (20.4)	15 (29.4)	0.283
Grade ≥ 3 thrombocytopenia	32 (30.5)	21 (38.9)	11 (21.6)	0.054
Grade ≥ 3 neutropenia	46 (43.8)	26 (48.1)	20 (39.2)	0.357
Grade ≥ 3 febrile neutropenia	9 (8.6)	5 (9.3)	4 (7.8)	>0.999
Grade ≥ 3 emesis	6 (5.7)	3 (5.6)	3 (5.9)	>0.999
Grade ≥ 3 stomatitis	2 (1.9)	0 (0.0)	2 (3.9)	0.234
Grade ≥ 3 diarrhea	14 (13.3)	6 (11.1)	8 (15.7)	0.491
Any documented treatment delay	61 (58.1)	34 (63.0)	27 (52.9)	0.298
Toxicity-related treatment discontinuation	7 (6.7)	4 (7.4)	3 (5.9)	>0.999

Note: Data are presented as *n* (%). Percentages were calculated within the relevant column. Grade ≥ 3 toxicities were recorded according to routine clinical documentation. Any documented treatment delay was defined as at least one recorded delay during second-line treatment. For toxicity-related treatment discontinuation, patients with a recorded discontinuation event were classified as having discontinued treatment due to toxicity. *p* values were calculated using the chi-square test or Fisher’s exact test, as appropriate.

**Table 4:** Univariable and multivariable Cox regression analyses for progression-free survival.

Variable	Univariable HR for PFS	95% CI	*p* Value	Multivariable HR for PFS	95% CI	*p* Value
Platinum-containing doublet vs. single-agent chemotherapy	0.53	0.36–0.80	0.002	0.46	0.29–0.72	<0.001
Age, per 10-year increase	1.04	0.83–1.31	0.720	1.26	0.98–1.61	0.071
Male sex	1.21	0.78–1.89	0.396	1.46	0.90–2.37	0.125
ECOG PS before second-line treatment	1.95	1.33–2.88	<0.001	1.74	1.16–2.59	0.007
Number of metastatic sites before second-line treatment	1.21	1.01–1.44	0.038	1.10	0.89–1.36	0.361
First-line atezolizumab exposure	1.22	0.81–1.83	0.337	1.26	0.81–1.97	0.305
Objective response to first-line treatment, CR/PR vs. SD	0.83	0.49–1.40	0.479	1.39	0.76–2.55	0.283
Platinum-free interval, per 30-day increase	0.83	0.67–1.02	0.075	0.91	0.73–1.14	0.428
Liver metastasis before second-line treatment	1.73	1.15–2.60	0.008	1.25	0.80–1.95	0.338
Brain metastasis before second-line treatment	1.54	1.03–2.31	0.035	1.46	0.92–2.32	0.111

Note: PFS, progression-free survival; HR, hazard ratio; CI, confidence interval; ECOG PS, Eastern Cooperative Oncology Group performance status; CR, complete response; PR, partial response; SD, stable disease. HR < 1 indicates lower risk of progression or death, whereas HR > 1 indicates higher risk. The reference group for treatment was single-agent chemotherapy. The reference group for sex was female. Objective response to first-line treatment was analyzed as CR/PR versus SD.

**Table 5:** Univariable and multivariable Cox regression analyses for overall survival.

Variable	Univariable HR for OS	95% CI	*p* Value	Multivariable HR for OS	95% CI	*p* Value
Platinum-containing doublet vs. single-agent chemotherapy	0.68	0.46–1.01	0.054	0.68	0.44–1.04	0.073
Age, per 10-year increase	0.99	0.79–1.26	0.965	1.18	0.92–1.51	0.201
Male sex	1.31	0.84–2.05	0.238	1.54	0.94–2.52	0.087
ECOG PS before second-line treatment	1.98	1.32–2.96	<0.001	1.81	1.20–2.75	0.005
Number of metastatic sites before second-line treatment	1.20	1.00–1.43	0.054	1.04	0.85–1.28	0.699
First-line atezolizumab exposure	1.23	0.81–1.86	0.333	1.17	0.75–1.82	0.486
Objective response to first-line treatment, CR/PR vs. SD	0.92	0.53–1.58	0.758	1.25	0.69–2.27	0.457
Platinum-free interval, per 30-day increase	0.86	0.70–1.06	0.157	0.99	0.79–1.23	0.925
Liver metastasis before second-line treatment	1.83	1.21–2.75	0.004	1.70	1.09–2.63	0.019
Brain metastasis before second-line treatment	1.67	1.11–2.52	0.014	1.53	0.96–2.44	0.075

Note: OS, overall survival; HR, hazard ratio; CI, confidence interval; ECOG PS, Eastern Cooperative Oncology Group performance status; CR, complete response; PR, partial response; SD, stable disease. HR < 1 indicates lower risk of death, whereas HR > 1 indicates higher risk. The reference group for treatment was single-agent chemotherapy. The reference group for sex was female. Objective response to first-line treatment was analyzed as CR/PR versus SD.

**Table 6:** Hazard ratio for platinum-containing doublet versus single-agent chemotherapy across eight analytic approaches.

Model	PFS HR (95% CI)	*p* Value	OS HR (95% CI)	*p* Value
1. Unadjusted	0.53 (0.36–0.80)	0.002	0.68 (0.46–1.01)	0.054
2. Full multivariable (10 covariates)	0.46 (0.29–0.72)	<0.001	0.68 (0.44–1.04)	0.073
3. Full multivariable + pleural metastasis	0.46 (0.29–0.73)	0.001	0.71 (0.46–1.10)	0.120
4. Parsimonious model	0.54 (0.35–0.82)	0.004	0.73 (0.48–1.10)	0.134
5. Propensity score as covariate	0.58 (0.38–0.88)	0.011	0.72 (0.47–1.11)	0.136
6. Stabilized IPTW	0.68 (0.38–1.20)	0.184	0.73 (0.46–1.16)	0.183
7. First-line responders only (*n* = 88)	0.55 (0.36–0.86)	0.008	0.72 (0.47–1.12)	0.147
8. ECOG PS 0–1 only (*n* = 91)	0.55 (0.36–0.85)	0.007	0.73 (0.48–1.11)	0.144

Note: All hazard ratios refer to platinum-containing doublet therapy versus single-agent chemotherapy; HR < 1 favors doublet therapy. Model 2 is the primary multivariable model reported in Table 4 and Table 5. The parsimonious model included ECOG performance status, liver metastasis, brain metastasis and platinum-free interval. IPTW denotes stabilized inverse probability of treatment weighting, with weights truncated at the 1st and 99th percentiles and robust variance estimation. CI, confidence interval; HR, hazard ratio; OS, overall survival; PFS, progression-free survival.

## Data Availability

The data supporting the findings of this study are available from the corresponding author upon reasonable request. The data are not publicly available because of restrictions relating to patient privacy.
